# Bioresonance therapy may treat depression

**DOI:** 10.25122/jml-2021-0008

**Published:** 2021

**Authors:** Daniela Muresan, Andreea Salcudean, Daniela Claudia Sabau, Cristina Raluca Bodo, Iosif Gabos Grecu

**Affiliations:** 1. Doctoral School of George Emil Palade University of Medicine, Pharmacy, Sciences and Technology, Targu-Mures, Romania; 2. Department of Ethics and Social Sciences, George Emil Palade University of Medicine, Pharmacy, Sciences and Technology, Targu-Mures, Romania; 3. Psychiatric Clinic 1, Mures County Hospital,Targu-Mures, Romania; 4. Psychiatric Clinic 2, Mures County Hospital, Targu-Mures, Romania; 5. Department of Psychiatry, George Emil Palade University of Medicine, Pharmacy, Sciences and Technology, Targu-Mures, Romania

**Keywords:** bioresonance therapy, depression, alternative medicine, electromagnetic waves

## Abstract

The aim of the study was to evaluate if bioresonance therapy can offer quantifiable results in patients with recurrent major depressive disorder and with mild, moderate, or severe depressive episodes by decreasing the level of depression due to the application of bioresonance therapy as independently or complementary treatment. The study included 140 patients suffering from depression, divided into three groups. The first group (40 patients) received solely bioresonance therapy, the second group (40 patients) received pharmacological treatment with antidepressants combined with bioresonance therapy, and the third group (60 patients) received solely pharmacological treatment with antidepressants. The assessment of depression was made using the Hamilton Depression Rating Scale, with 17 items, at the beginning of the bioresonance treatment and the end of the five weeks of treatment, aiming to decrease the level of depression. The study identified the existence of a statistically significant difference for the treatment methods applied to the analyzed groups (p=0.0001), and we found that the therapy accelerates the healing process in patients with depressive disorders. Improvement was observed for the analyzed groups, with a decrease of the mean values between the initial and final phase of the level of depression, of delta for Hamilton score of 3.1, 3.8 and 2.3, respectively. We concluded that the bioresonance therapy could be useful in the treatment of recurrent major depressive disorder with moderate depressive episodes independently or as a complementary therapy to antidepressants.

## Introduction

Depressive disorders involve feelings of sadness, emotional void, irritability and are accompanied by somatic and cognitive changes that significantly affect the individual’s ability to function [1].

Depressive disorders comprise affective disorders with disruptive mood dysregulation disorder, major depressive disorders, including a major depressive episode, persistent depressive disorders or dysthymia, premenstrual dysphoric disorder, substance or drug-induced depressive disorders, depressive disorders caused by a medical condition, other depressive disorders, and nonspecific depressive disorders. Depending on the number and severity of the symptoms, a depressive episode can be classified as mild, moderate, or severe [2].

This negative emotional state can persist for a short period or a longer period, with mild, moderate, or severe intensity, which can seriously damage one’s health. At worst, depression can lead to suicide. Nearly 800,000 people die each year from suicide [3]. In 2017, over 300 million people, the equivalent of 4.4% of the world’s population of all ages, suffered from depression, with an increase of over 18% between 2005 and 2015. Depression is more common among women than men. Worldwide, the prevalence of depression varies according to age, exceeding 7.5% among women aged 55–74 and 5.5% among men in the same age group [4].

In Europe, one in twenty people currently suffers from depression and one in four will go through a depressive episode at some point in their lives [5]. In Romania, approximately 5% of the population suffers from a form of diagnosed depressive disorder, the subjects in question being under medical treatment [6]. Health experts estimate that, by 2030, depression will become the most significant contributor to the global burden of mental and behavioral disorders. It currently occupies second place in the ranking of diseases worldwide, following cardiovascular diseases [7]. Although there are known and effective treatments for mental health problems, between 76% and 85% of people from low-income and middle-income countries do not receive any treatment for their condition [8].

In the constant effort to find solutions, the scientific community addressed the human energy-generating processes. In moments of stress, sadness, and prolonged despair, the energetic balance is broken, causing a disruption in the inner flow of energy, a disharmony, a disturbance or even a blockage, which leads, progressively at first, to preclinical effects, accompanied by informational, energetic, biophysical and biochemical changes, and then clinically manifest symptoms appear before the disease settles with the whole clinical picture [9].

Since 1970, bioresonance therapy has been used successfully in various ailments by many practitioners around the world, being integrated into alternative medicine. Bioresonance therapy is a less familiar method of therapy, and most patients find it difficult to opt for, while others refuse this type of complementary therapy.

In this study, we aspired to analyze if a new method of therapy, independently or complementary to drug treatment, is suitable and useful for patients diagnosed with recurrent major depressive disorder and with mild, moderate, or severe depressive episodes by decreasing the level of depression quantified with the Hamilton scale. We aimed to verify the null hypothesis (H0) for each group: the applied therapy does not accelerate the healing process in patients with recurrent major depressive disorder or those with a mild, moderate, or severe depressive episode. The alternative hypothesis (H1) was: the applied therapy accelerates the healing process in patients with recurrent major depressive disorder and those with a mild, moderate, or severe depressive episode.

## Material and Methods

We included in the study patients diagnosed with recurrent major depressive disorder with a mild, moderate, or severe depressive episode using the criteria listed in the Diagnostic Manual of Mental Disorders Five (DSMV). We excluded the patients that had suicidal attempts noted in their medical history, patients with a peacemaker, and pregnant women. We selected the patients from the Mureș County Clinical Hospital, the Psychiatry Clinic I, Targu-Mures, Romania, and the specialized outpatient clinic and in the Terapia Ultramed Bioresonance Therapy Practice within Terapia Ultramed Clinic of Targu-Mures, Romania.

The study was a retrospective study and was conducted between October 2017 and October 2018. Written consent was obtained from participants after they were informed about the study and its implications. Consent was also obtained from appropriate Romanian authorities. Data protection was ensured. The study was approved by the Institutional Ethics Committee of the Mures County Hospital from Targu Mures under the number 16462/16.10.2017. The analyzed group consisted of women and men from different social backgrounds, aged between 18–89 years. We split the participants into three groups: group 1 consisted of 40 patients (31 women and 9 men). Seventeen women had mild depressive episodes, and 14 women had moderate depressive episodes. Within the male category, 5 men had been diagnosed with a mild depressive episode and 4 men with a moderate depressive episode. They received solely bioresonance therapy. Group 2 consisted of 40 patients (33 women and 7 men). Two men had mild depressive episodes, 32 women and 5 men moderate depressive episodes, and one woman had been diagnosed with severe depressive episodes. They received pharmacological treatment with antidepressants and bioresonance therapy. Group 3 consisted of 60 patients (31 were women and 29 men). All study participants in this group had been diagnosed with moderate depressive episodes. They received only pharmacological treatment with antidepressants. The detailed demographic structure of groups is detailed in Table 1.

**Table 1.** Demographic aspects of the study groups.

	Group 1	Group 2	Group 3	P-value
**Female sex**	77.50%	82.50%	51.70%	0.0001
**Age (years) mean±sd**	56.43	64.048	73.28	0.0001
**Marital Status**				
Married	50.00%	57.50%	51.70%	0.56
Divorced	30.00%	20.00%	11.70%	0.003
Unmarried	20.00%	10.00%	20.00%	0.09
Widower	0.00%	12.50%	16.17%	0.0003
**Studies**				
Primary	0.00%	0.00%	20.00%	0.0001
Middle school	0.00%	12.50%	11.70%	0.001
High scool	30.00%	25.00%	26.70%	0.70
Secondary education	12.50%	12.50%	3.30%	0.03
Vocational school	5.00%	35.00%	16.60%	0.0001
Higher education	52.50%	15.00%	21.70%	0.001
**Environment**				
Rural	35.00%	47.50%	38.30%	0.18
Urban	65.00%	52.50%	61.70%	0.18

### Demographic aspects of the study groups

Group 1 and group 2 received bioresonance therapy independently and complementary to antidepressant medication, respectively. For all patients, we aimed to decrease the level of depression, within a period of maximum 2 months, from the first session to the fifth session.

The bioresonance treatment consists of connecting the patient to a Mora Nova bioresonance device, with the help of two-dimensional electrodes for hands and feet. The device automatically records the values of the eight quadrants obtained at the first and second measurements during a single treatment session. During the measurement of the values, the device emits a sound of different intensity, depending on the values obtained [10].

The duration of a session applied to the patients in the study was about 20 minutes, with a weekly frequency. The therapy was individualized according to the biorhythm of the patient and incorporated high and low potencies. Through biorhythm, the therapeutic method selected from the device software regulated and controlled the patient’s oscillating information, as well as his ability to adjust, in order to initiate precise target healing processes.

Endogenous therapy is included in the principle of basic bioresonance therapy with the Mora Nova device and is considered the fundamental principle of bioresonance therapy at the physical level. This type of therapy involves the destructive inference or the so-called “overlapping extinction” of rigid, isolated vibrations, considered pathological vibrations with themselves. In this way, they seem to be integrated into the flexible and dynamic vibrational composition of human beings through the processes of self-regulation. The physiological blockages correlated with the “rigid” vibrations dissolve later. According to these hypotheses, “pathological vibrations” are correlated with the disease on an electromagnetic plane. A weak electromagnetic interaction has a physiological consequence due to the informative catalytic effect because they are weak interactions in an initially fragile situation. The energy needed to carry out the program must be provided by the living system itself through the acupressure points of the lower and upper limbs. Bioresonance therapy also promotes the individual’s potential for self-healing [10]. During therapy, the patient must not carry a smartphone, other electronic devices, metals in contact with the skin, or mechanical devices. Before and after each treatment session, the electrodes were cleaned with 70% alcohol [11]. The Mora Nova device is an electromagnetic transceiver, which must be connected to a source of electricity and is equipped with a backup battery, with a frequency between 0.1 Hz and 480,000 Hz and with a frequency filter from 1 Hz up to 500,000 Hz [12].

We used the Hamilton Scale composed of 17 items (Ham-D-17) for assessing depression, the questionnaire being composed of questions rated with 0–2 or 0–4 points, 4 being the most severe. The total score of the HAM-D-17 scale varies between 0–52 points. The scale measured individual depressive symptoms and their general severity, reflected by a final score, which indicated the degree of depression. A score >25 pointed to severe depression, a score between 18–24 showed moderate depression, a score between 8–17 showed mild depression, and a score <7 was considered normal.

**Figure 1.**
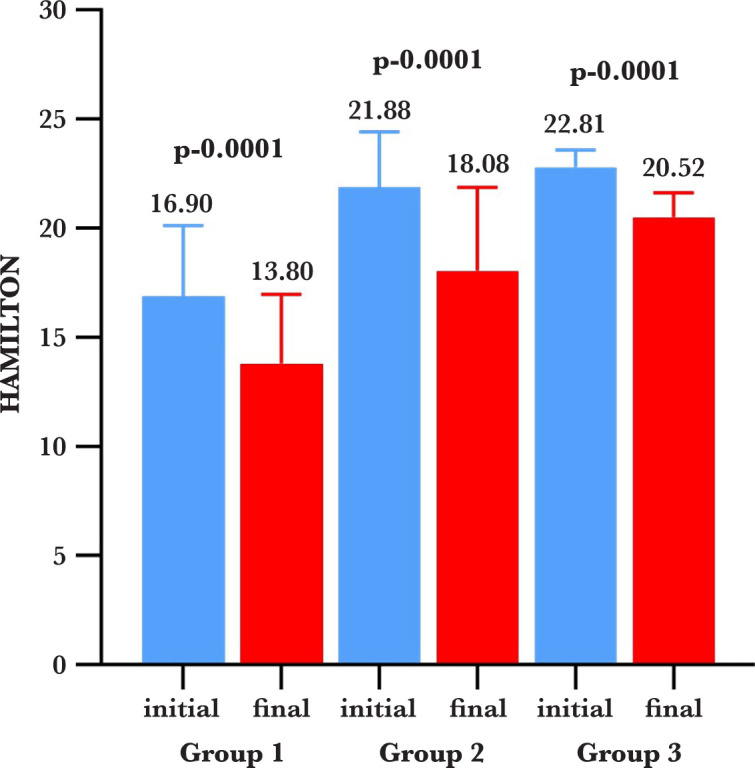
Comparison of the Hamilton Scale between the initial and the final sessions for each group.

For calculating the statistical indicators, we used the functions of the Excel program of Microsoft Office, the Google Docs package, and the Tukey test. We assessed the change in depressive symptoms from the first to the last session of therapy, using the Hamilton Depression Rating Scale with 17 items, the bioresonance therapy being applied once a week. For group 3, we assessed the change in depressive symptoms after five weeks of taking antidepressants. The Hamilton score is shown in Figure 1. A comparison of the Hamilton Scale between the initial and the final sessions was made for each group.

## Results

After conducting the above-mentioned steps, we obtained a significant value (p=0.0001) for group 1, so the initial mean values of the Hamilton scale were higher than the final mean values. We presented the results as a box-plot type graph, expressing the mean and standard deviation, highlighting the decrease of the mean values between the initial and the final phase, delta being -3.10.

For group 2, we obtained a statistically significant value as well (p=0.0001), and the initial mean value of the Hamilton scale was higher than the final one, the decrease of the mean values between the initial and final phase being -3.80. This is the highest difference obtained for the groups. A statistically significant value (p=0.0001) was obtained for group 3 as well; the initial mean value of the Hamilton scale was higher than the final one, the decrease of the mean values between the initial and final phase being -2.30, the smallest diminution across the groups. Tukey’s multiple comparison test was used to compare the results obtained for each group, and the results are presented in Table 2.

**Table 2.** Comparative inferential statistics of the Hamilton scale.

Tukey's multiple comparisons test	Mean dif.	95% CI of dif.	Significance	Summary
**Group 1 (initial vs. final)**	3.100	1.482 to 4.718	Yes	****
**Group 2 (initial vs. final)**	3.800	2.182 to 5.418	Yes	****
**Group 3 (initial vs. final)**	2.300	0.9791 to 3.621	Yes	****

## Discussion

The current study intended to identify whether bioresonance therapy has quantifiable results in the treatment of patients diagnosed with recurrent major depressive disorder or with a mild, moderate, or severe depressive episode.

The specialized literature presents several studies in which bioresonance therapy is successfully used in the case of various pathologies. To our knowledge, no study determined and analyzed an objective evaluation for its effectiveness in the treatment of depression, as a standalone therapy or as a complementary therapy.

In 2018, a controlled clinical study was performed in Russia on 60 patients who were high-performance athletes affected by excessive physical exertion and addressed the regulation of systolic blood pressure, heart rate, and the reduction of stress by restoring the psycho-emotional balance; the intervention group showed better results compared to the placebo group [13]. Bioresonance therapy can significantly improve gastrointestinal disorders, as presented in a randomized controlled study performed in Germany on 20 people with psychosomatic diseases and gastrointestinal disorders [14].

An observational pilot study that included eight patients with lymphedema and lower limb lipedema demonstrated that bioresonance therapy in lymphedema and lipedema was effective, leading to reduced edema, relieving symptoms and improving lymphatic drainage, without side effects [15]. A German prospective controlled clinical study performed on 190 smokers has proven that bioresonance therapy was effective in quitting smoking and has no side effects [16].

In recent years, bioresonance therapy has proven to be a feasible treatment in several pathologies, both complementary to classical therapy or used independently. After measuring the level of depression using the Hamilton Depression Rating Scale for each group, we noticed differences between the initial mean values versus the final mean values in the first session and the fifth session. In group 1, in which the patients received only bioresonance therapy, we obtained a statistically significant value (p=0.0001); the mean value was higher at the initial stage compared to the final stage, which indicated a decrease in the mean values of delta (-3.10) between the initial and the final phase. In group 2, patients were receiving antidepressants and bioresonance therapy (combined treatment). There was a statistically significant value (p=0.0001); the average was higher at the initial stage compared to the final stage, which indicated a decrease in the mean values between the initial and final phases of delta (-3.80), this being the most important difference among the three analyzed groups. In group 3, there was a statistically significant value as well (p=0.0001); the average was higher at the initial stage compared to the final stage, with a decrease of the mean values delta being (-2.30) between the initial and final phases. This was the smallest decreased amount the groups but still noteworthy.

Among the patients in the first group, after applying the five bioresonance therapy sessions, 4 men and 10 women reported reduced depressive episodes, from a moderate to a mild depressive level. In the second group, after applying the five bioresonance therapy sessions combined with drug treatment, 1 woman went from a severe depressive episode to a moderate depressive episode, and 2 men and 6 women went from a moderate depressive episode to an episode of mild depression. A male patient suffering from mild depressive episodes recovered completely. Among the study participants in the third group, both women and men remained with a moderate depressive episode.

## Conclusion

The study results confirmed that bioresonance could improve the level of depression assessed with the Hamilton Depression Rating Scale with 17 items in patients suffering from depression, independently or as a complementary therapy to antidepressant medication.

## Acknowledgments

### Ethical approval

The approval for this study was obtained from the Ethics Committee of the Mures County Hospital, Targu Mures, Romania (approval no. 16462/16.10.2017).

### Consent to participate

Informed consent was obtained from the participants.

### Conflict of interest

The authors declare that there is no conflict of interest.
